# Effects of electric field on micro-scale flame properties of biobutanol fuel

**DOI:** 10.1038/srep32938

**Published:** 2016-09-09

**Authors:** Tao Xu, Qinglin Chen, Bingjian Zhang, Shushen Lu, Dongchuan Mo, Zhengguo Zhang, Xuenong Gao

**Affiliations:** 1 Guangdong Engineering Technology Research Center for Petrochemical Energy Conservation, School of Chemical Engineering and Technology, Sun Yat-sen University, Guangzhou 510275, China; 2 Key Laboratory of Enhanced Heat Transfer and Energy Conservation (Ministry of Education), School of Chemistry and Chemical Engineering, South China University of Technology, Guangzhou 510640,China

## Abstract

With the increasing need of smaller power sources for satellites, energy systems and engine equipment, microcombustion pose a potential as alternative power source to conventional batteries. As the substitute fuel source for gasoline, biobutanol shows more promising characteristics than ethanol. In this study, the diffusion microflame of liquid biobutanol under electric field have been examined through in-lab experiment and numerical simulation. It is found that traditional gas jet diffusion flame theory shows significant inconsistency with the experimental results of micro scale flame in electric field. The results suggest that with the increase of electric field intensity, the quenching flow rate decrease first and increase after it reach its minimum, while the flame height and highest flame temperature increase first and drop after its peak value. In addition, it was also observed that the flame height and highest temperature for smaller tube can reach its maximum faster. Therefore, the interaction between microscale effect and electric field plays a significant role on understanding the microcombustion of liquid fuel. Therefore, FLUENT simulation was adopted to understand and measure the impacts of microflame characteristic parameters. The final numerical results are consistent with the experimental data and show a high reliability.

In the last few years, miniaturization of energy systems and engine equipment, such as microsatellites and micro aerial vehicles, attracts many research efforts^1^,^2^,^3^,^4^. The need of micro-power sources to power up these systems has dramatically increased. Since the energy density of typical hydrocarbon fuels is about 100 times higher than that of the most advanced batteries, hydrocarbon fuel-based microcombustors are regarded as the optimal substitution of traditional batteries^5^,^6^,^7^,^8^. Since the characteristics of diffusion flames is the critical concern in the development of microscale combustion systems^9^, many studies have investigated the characteristics of gaseous fueled flames^10^,^11^,^12^,^13^. However, very few studies focus on analyzing micro diffusion flames of liquid fuels^14^,^15^,^16^. Comparing to gaseous fuels, liquid fuels have higher energy density and easier to be transported. Although liquid fuels show a great potential for microcombustion systems, their combustion is more complicated than gas combustion because of gasification. Also, it is challenging to maintain the sustainable combustion in a microcombustor, because of higher heat losses and surface to volume ratio could lead to suppressing ignition and quench the reaction^4^. Therefore, it is necessary to investigate the mechanisms of liquid fuel microcombustion for better system design and parameter optimization.

Many researchers have studied the characteristics of diffusion microflames. Ban *et al*.^17^ found that the gravity effect on the structure of diffusion microflame with infinite space was negligible. Nakamura *et al*.^18^ discovered that minimum limits and power of diffusion microflame were about 1 mm^3^ and 1 W respectively. Matta *et al*.^19^ measured the quenching limits of diffusion microflames and found that the experimental values agreed with the predicted values based on laminar jet diffusion flame theory. Catalytic effect can maintain a stable combustion of hydrocarbon gas fuel in micro scale burner^20^,^21^. D. G. Norton *et al*.^22^ found that wall thermal conductivity and wall thickness were important factors for ignition and flame stability. Cheng *et al*.^23^ concluded that Roper’s model could ideally predict the flame height and quenching flow of methane microflames. Kyritsis *et al*.^24^ and Pham *et al*.^25^ proposed a mesoscale catalytic combustor based on multiplexed electrosprays for liquid fuel dispersion and a liquid film combustor. As expected, the microflame of liquid fuel is unstable and easier to extinguish. At the same time, Katuoke electric wind effect exists in the process of combustion reaction in the electric field and improves the efficiency of combustion^26^,^27^,^28^. Therefore, the electric field effect shows promising characteristics to improve the combustion stability and efficiency of liquid fuel microflames.

Recently, Xu *et al*. conducted series of experimental and numerical studies on laminar flow diffusion microflames and microcombustor of liquid ethanol^29^,^30^,^31^,^32^. Built upon previous research, this study aims to investigate the mechanism of electric field and microscale effect on liquid fuels microcombustion. Comparing to previous research on ethanol, biobutanol is examined because of its advantages in caloric value, cold start, safer service, erosion and abrasive wear. In this research, the impacts of flow rate, electric field and microscale on the diffusion microflames of liquid fuels are analyzed through in-lab experiment and numerical simulation.

## Experiment and Simulation

### Experimental Design

**Figure 1:**
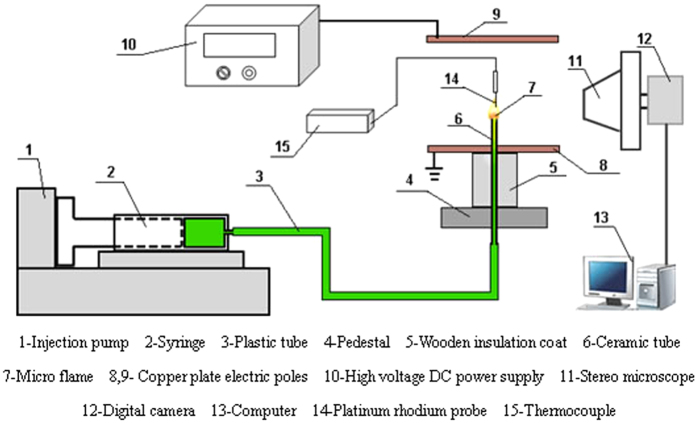
Schematic diagram of experimental set-up.

Figure 1 illustrates a schematic diagram of the experimental set-up for diffusion microcombustion tests. Two ceramic tubes with inner diameter of 0.4 mm and 1.0 mm and outer diameter of 2.0 mm are selected as combustors. Then tubes are installed inside an inner round hole with wooden insulation coat to reduce heat loss and then placed vertically in the pedestal with four adjustable bolts. The microinjection pump (TS2-60, Baoding Longer Precision Pump Co., Ltd., China) with the fuel flow rates from 1 μL/h to 63 ml/h with a deviation of less than 1% is installed to transport fuel. The fuel characteristics of biobutanol are listed in Table 1. The stereo microscope is connected to a high resolution digital camera (ProgRes C^12^, Eyelike Instruments, Germany) and a computer to capture the microflame data. The microflame structure is measured by Origin software with allowable errors of ±0.01 mm. An S-type thermocouple (working temperature between −50 °C to 1300 °C; error of ±1 °C) with a platinum-rhodium probe is linked to an Agilent 34970 A data acquisition unit to monitor the temperature variations of the microflame. A high voltage DC power supply (DW-P103-1 AC, Tianjin Dongwen High Voltage Power Supply Co., Ltd., China) with two square copper plate(10 mm × 10 mm × 2 mm) electrodes is utilized to generate electric field system. The High voltage DC power source is able to supply direct voltages from 0 V to 10000 V. One of copper plates has 2 mm hole in the center is set as the positive electrode, which is installed horizontally on the pedestal, during the experiment, the ceramic tubes can move through the hole. The vertical distance from the outlet of tubes to the positive electrode is 8 mm. The other copper plate, which is regarded as negative electrode, has a 40 mm distance away from the positive electrode. Since the distance is fixed, then the intensity of electric field can be ajusted by varying the supply voltage. The electric field intensity is determined by uniform electric field equation:


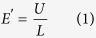


Where 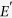 represents the electric field intensity; *U* is the applied voltage; *L* is the electrode distance. The measurement error *ΔU* and *ΔL* are ±1 V and ±1 mm.

The error of electric field intensity 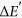
is calculated by the transfer function:


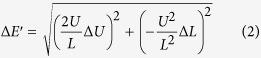


### Simulation method

**Figure 2:**
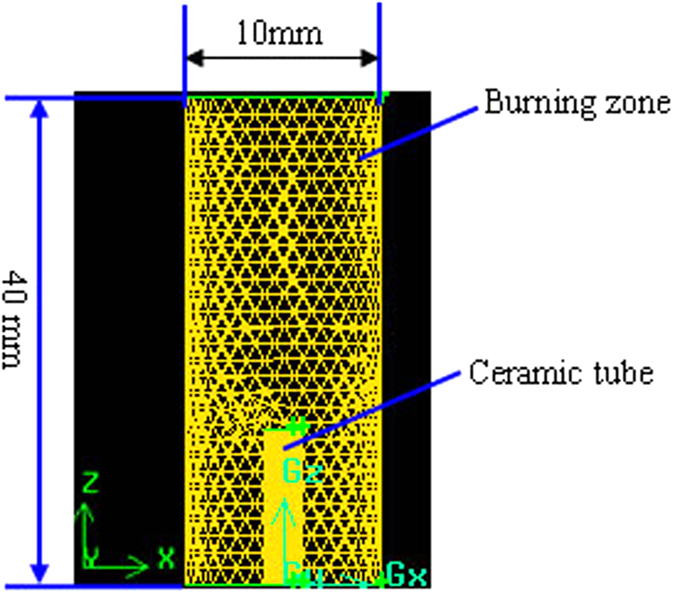
Grid system for combustion.

A computational fluid dynamic (CFD) software, Fluent, is used for the numerical analysis. User-defined Scalar (UDS) package in Fluent 6.3 is adopted to solve electric fluid dynamics problems. Because the micro-scale combustion have special properties, such as a very short residence time, increased surface area volume ratio, significant viscous effect, and large quantity of heat loss and so on, it is very important to select 3 D model for the accurate predictability of combustion process. The suited 3 D model as shown in Fig. 2 includes burning zone outside tube, tube wall and flowing zone inside tube. Combining the characteristic of laminar flow diffusion combustion, a 3D cylinder combustion model with height of 40 mm and diameter of 10 mm is created. The height of tube 1 and tube 2 are set as 8 mm in the experiment. The models are created and meshed by GAMBIT, a pre-processing software package in Fluent. After a test on the mesh number effects, 208 351 and 59 950 hexahedral elements were generated for tube 1 and tube 2. Then, the model was exported to Fluent with boundary conditions and material properties set to default. The interface between the tube and fuel was treated as a coupled wall.

Different from gaseous fuel, the combustion of liquid fuel simulation includes more complicated droplet evaporation, mixture and combustion. Following assumptions had made to simply the model:

1. The density change due to fuel and air was negligible.
2. The specific heat and thermal conductivity of tube was constant.
3. The heat dissipated through radiation was neglected.
4. The combustion products were just CO_2_, CO and H_2_O.
5. The combustion process was simplified to the following two-step reaction:

C_4_H_9_OH + O_2 _→ CO + H_2_O

CO + O_2_ → CO_2_

Based on above assumptions, the laminar flow diffusion combustion of liquid fuel must consistent with the mass conservation equation, momentum conservation equation, energy conservation equation, and component mass conversion equation^32^.

The mass conservation equation can be written as follows


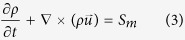


In Eq. 3, *ρ* is the density, 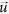 is the velocity, and the source *S*_*m*_ is the mass added to the continuous phase from the dispersed second phase and any user-defined sources.

The momentum conservation equation is described by


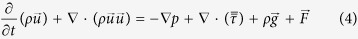


Where *p* is the static pressure, 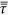 is stress tensor, and 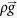 and 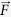 gravitational body force and external body forces, respectively.

Then, the energy equation can be solved as


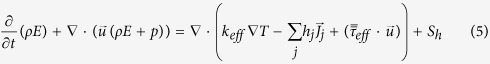


In Eq. 5, 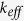 is the effective conductivity, 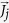 is the diffusion flux of species *j*, 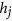 is the sensible enthalpy of species *j*, 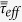 is the effective stress tensor, E can be expressed as 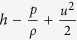, and *S*_*h*_ includes the heat of chemical reaction and any other volumetric heat sources to be defined.

The component equation follows Eq. 6 during biobutanol burning.


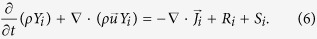


Where *Y*_*i*_ is the mass fraction of species *i*, *R*_*i*_ is the net rate of production of species *i* by chemical reaction, and 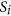 is the rate of creation by addition from dispersed phase plus any user-defined sources.

The gasification model of liquid fuel has never considered previously because researchers have concentrated on trying to study the microscale combustion properties of gaseous fuel. It can be expressed through calculating heat change of liquid droplet as follow:


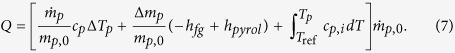


Where, 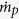, 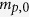, 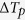, 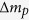, 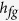, 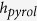, 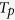, 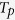, 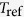, 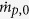 are represented by the average mass rate, initial mass, temperature variation, mass rate variation, vaporization latent heat of vaporization, devolatilization heat, final temperature, reference temperature and initial mass rate, respectively.

The voltage control equation was expressed as follow:


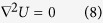


The initial and boundary conditions were as follows:

1. Mass flow inlet and pressure outlet are selected.
2. Initial fluid in burning zone is defined as air whose gauge pressure and temperature are set to 0 Pa and 300 K. The mass fraction of oxygen in the air is 22%.
3. Solid zone is defined as ceramic tube whose density and thermal conductivity are 2872 kg/m^3^ and 1.75 W/m·K respectively.

Combining with these boundary conditions, the governing equations were solved through the finite element method.

Given the low-speed incompressible flow, the pressure-based solver is more suitable comparing to the density-based solver. The parameters of the flow motion in the solution method and solution control retained as default, with the second-order up-wind difference scheme for energy and first order implicit transient formulation. Both boundary slip and droplet radiation heat transfer were considered for the process of microscale combustion from our previous study^32^. The residual for energy was set to 10^−6^ to make sure the convergence of energy equation.

## Results and Discussion

### Numerical Simulation Validation

Based on previous study^32^, the same characterization method for flame height was employed. The brighter flame is the higher the temperature. The flame temperature contour chart of numerical simulation is considered as flame structure. ***H*** is defined as the height of the flame.

**Figure 3:**
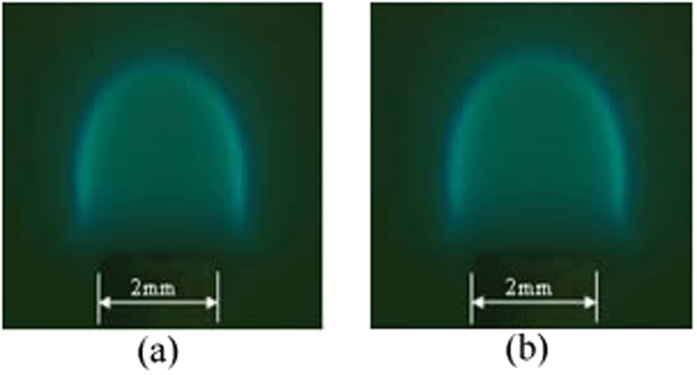
Experimental microflame structure with flow rate 0.9 ml/h for tube 1. (**a**) 0 V; (**b**) 4000 V.

**Figure 4:**
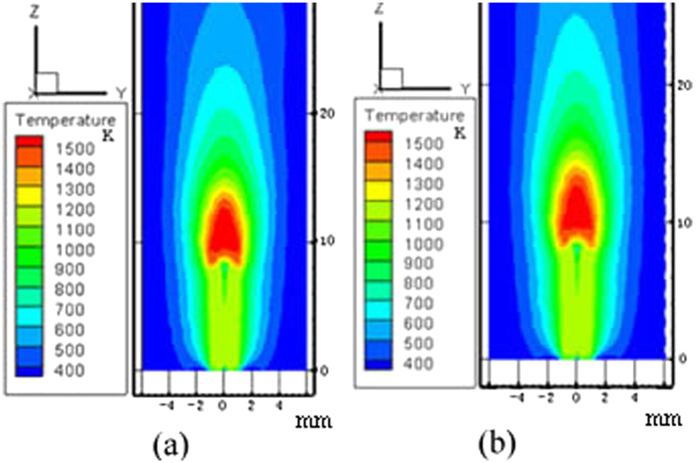
Flame temperature field of numerical simulation with flow rate 0.9 ml/h for tube 1. (**a**) 0 V; (**b**) 4000 V.

**Figure 5:**
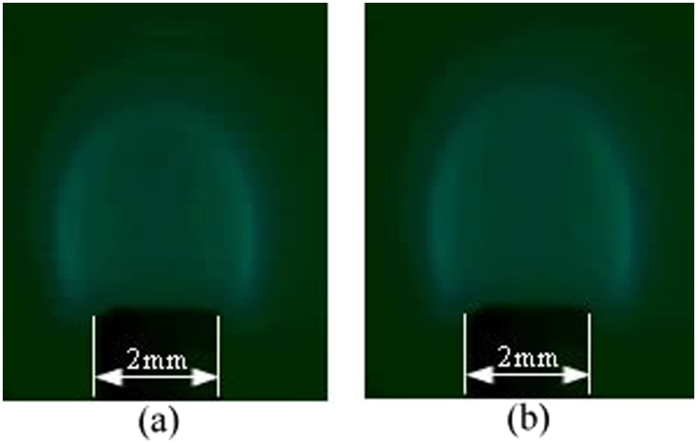
Experimental microflame structure with flow rate 1.8 ml/h for tube 2. (**a**) 0 V; (**b**) 4000 V.

**Figure 6:**
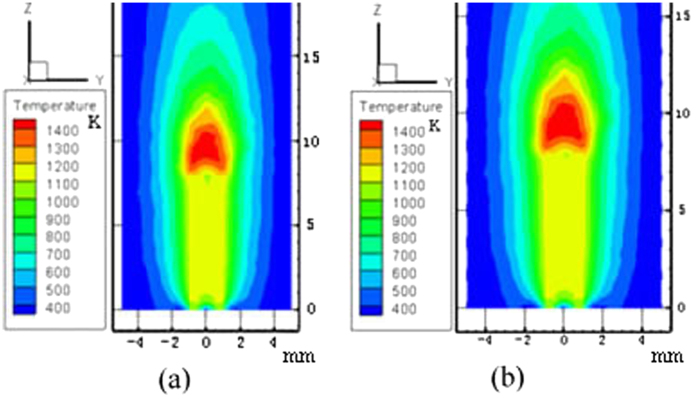
Flame temperature field of numerical simulation with flow rate 1.8 ml/h for tube 2. (**a**) 0 V; (**b**) 4000 V.

For both tube 1 and tube 2, the experimental microflame structure and temperature field of numerical simulation are shown in Figs 3, 4, 5, 6, respectively. ***T***_***A***_ refers to the temperature of the reference point, where is 2 mm away from the micro ceramic tube orifice center. Simulated values with different DC voltage are compared with experimental results based on their deviation of flame characteristics to quantify the impact. The comparison results are listed in Table 2. With the numerical simulation results of the flame characteristics, we found:

1. The values of flame characteristics, such as which include fame height and temperature of the reference point, in numerical simulation are greater than those in experiment. One possible explanation is that the CFD model does not account for ambient heat losses. Another reason could be the effect of flow air in the experimental environment.
2. It can be observed that deviations between experiment and simulation of tube 1 are 9.13% for ***H*** and 1.94% for ***T***_***A***_; similarly, for tube 2, maximum deviations are 7.31% for***H*** and 2.24% for ***T***_***A***_. Since, the diffidence is within 10% threshold, the simulation results are relatively consistent with the experiment results.

### Effect of microflame quenching

**Figure 7:**
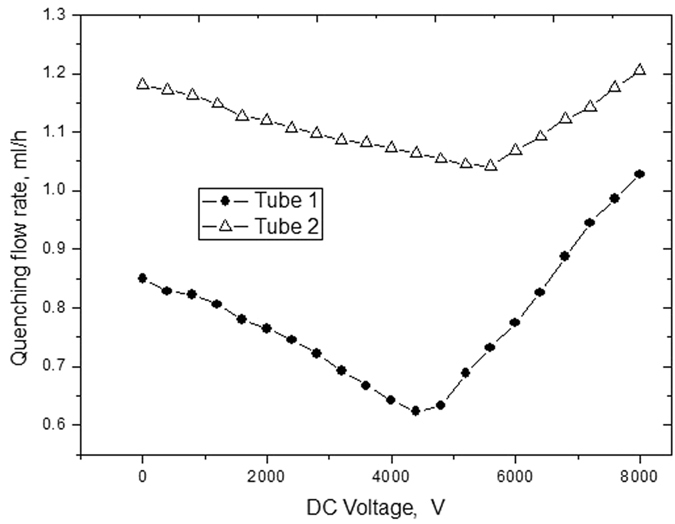
Flame quenching flow rate variation with different DC voltages.

It is crucial to determine the quenching flow rate of stable combustion for the design of micro burner with liquid fuel. To capture the quenching flow rate through experiment involves two steps: first, obtain the stable flame with suitable DC voltage; second, decrease the flow rate until the quenching. The flame quenching flow rate variation with different DC voltages in our experiment is shown in Fig. 7. It is obvious that with the increase of DC voltage, the quenching flow rate for tube 1 and 2 will decrease first, and then increase gradually after reaching its minimum value. According to the experiment results, tube 1 reach its minimum quenching flow rates 0.623 ml/h at 4400 V and tube 2 reach its minimum quenching flow rate when DC voltage is 5600 V.

The earliest researches discuss mechanism between electric field and flame can be traced back to 20’s of last century. After that the ion wind theory becomes popular in analyzing the impact of electric field on stable combustion, pollutant discharge and adiabatic combustion^33^,^34^,^35^,^36^,^37^. Based on the ion wind theory, the findings in our experiment can be explained. At the beginning of the combustion, the ion wind can improve combustion rate and liquid vaporization, which leads to decreased flame quenching flow rate. Then later, excessive ion wind will shorten sharply burn time of fuel, which causes the increase of flame quenching flow rate. Comparing tube1 with tube 2, the quenching flow rate for smaller tube reaches minimum value much faster within electric field. This implies that microscale effect and electric field affect the flame quenching flow rate.

### Effect of microflame height

**Figure 8:**
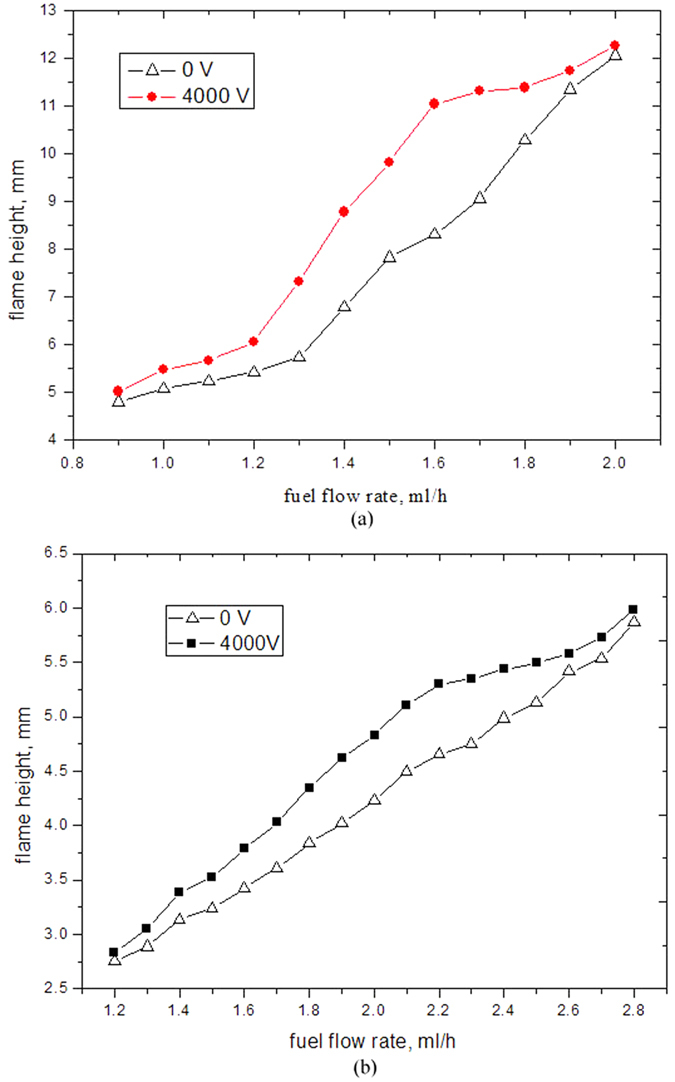
Flame height variation with flow rate. (**a**) tube 1; (**b**) tube 2.

As shown in Fig. 8, when the liquid fuel evaporated inside the tubes and flowed out form the steady diffusion flame, their flames have similar characteristics to gas fuel flames. Thus, the traditional gas jet flame theory of Roper^38^ was chosen as a comparison in this study. The flame heights were measured directly from the numerical simulation results in Fig. 8. Traditional gas jet diffusion flame theory suggests that the flame height is directly proportional to flow rate and independent of the tube diameter. The flame height of tube 2, who has a larger inner diameter, is independent of electric field and increases almost linearly with the fuel rate increases. However, the flame height variation in tube 1, who has a smaller inner diameter, shows a completely different trend.

In the systems without electric field, flame height rises slowly if the flow rate is below 1.3 ml/h and flame height rises rapidly if the flow rate is beyond 1.3 ml/h. With 4000 V electric field, flame height increases slowly when flow rate is below 1.2 ml/h or beyond 1.6 ml/h and flame height increases rapidly when flow rate is between 1.2 ml/h and 1.6 ml/h, which do not agree with laminar gas jet flame theory. When flow rates are low, the flame height increase for smaller tube is slower, because of the capillary force hinder the liquid biobutanol evaporation and combustion. When flow rates are higher, the more biobutanol is burnt to release more heat, and the temperature of ceramic tube becomes higher. This effect weaken the capillary force action and make more combustion particles drag from micro scale tube, therefore, the flame height become larger obviously.

As shown in Fig. 8, electric field has an obvious impact on the flame height. When flow rate is same, the flame height with 4000 V electric field is larger than that without electric field for both tube 1 and tube 2. According to katuoke electric wind effect principle, surplus positive ions exist in the outer layer of flame and negative ions exist in the inner zone, electric field can accelerate positive ions to move forward nozzle distance and make the flame longer. When the flow rate beyond threshold, the flame heights with electric field and without electric field are close to each other because the electric field action force has already reached its limitation. Therefore, the combined mechanisms of microscale effect and ion wind for the smaller tube play a more important role in the liquid fuel micro combustion in electric field and affect the diffusion flame behavior.

### Effect of microflame temperature

Given constant fuel volume and pressure, part of combustion heat is released to surrounding environment and the rest will heat the fuel, tube and combustion products. If heat loss is ignored, the flame will research the highest temperature (or adiabatic combustion temperature). Since electric field has such effect on the flame temperature, we take the highest temperature in to our consideration and overlook the influence of heat loss in the numerical simulation.

**Figure 9:**
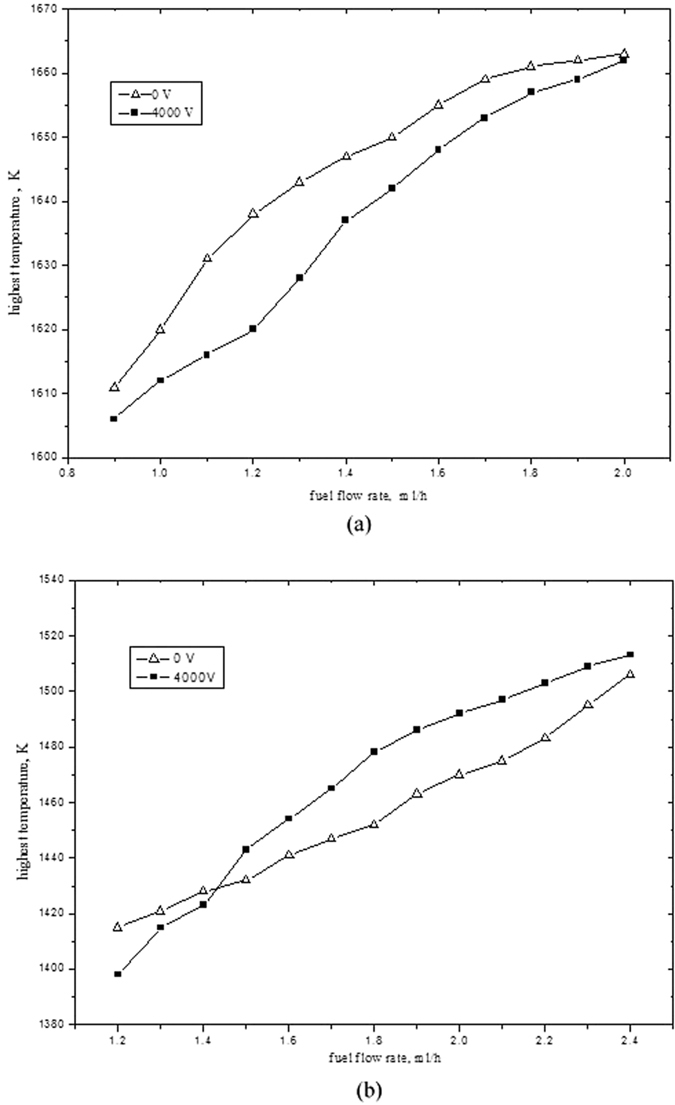
Relationship between highest temperature and flow rate. (**a**) tube 1; (**b**) tube 2.

Although the highest temperature of the flame is difficult to be directly measured by experimental tools, it can be shown in the flame temperature contour chart of numerical simulation as observed in Figs 4 and 6. The interrelationship between highest temperature and flow rate is presented in Fig. 9. The highest temperature increases almost linearly with the increase of liquid biobutanol flow rate. More fuel combustion can release more heat, which will make the temperature of ceramic tube go up to overcome capillary force and improve the evaporation rate of biobutanol, the burning will become more sufficient and result in higher the flame temperature. Besides that, electric field and flow rate have a different impact on the highest temperature in different inner diameter tube. The highest temperature for tube1 with 4000 V electric field is higher than that without electric field. For tube 2, if flow rate is beyond 1.4 ml/h, the highest temperature with 4000 V electric field is also higher than that without electric field; while the highest temperature with 4000 V electric field below flow rate of 1.4 ml/h, the higher temperature is lower than that without electric field. The main reason is that the ion wind of electric field can drag a large amount of air into combustion zone to degrade the temperature of flame when the density of gaseous biobutanol under the condition of lower flow rate is too lower. Through comparing the flame temperature properties of two tubes, it can be found that the fuel in smaller tube can completely burn, so that ion wind of electric field will destroy this combustion balance and degrade the flame temperature. However, the ion wind of electric field will improve the combustion efficiency, because of incomplete combustion for larger tube.

**Figure 10:**
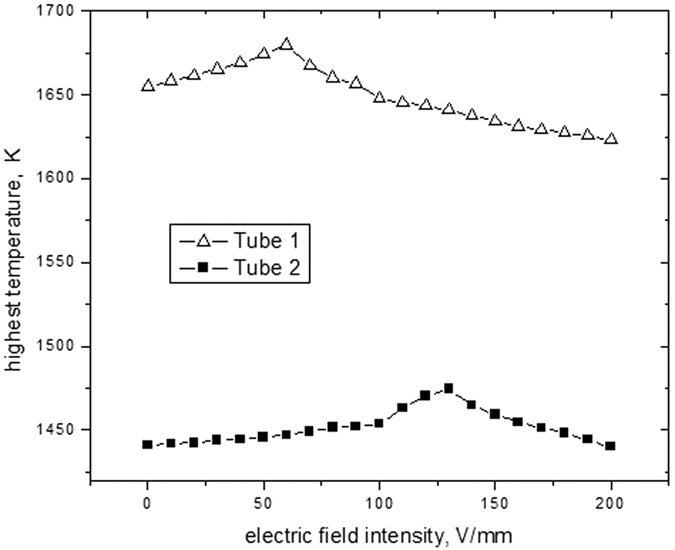
Highest temperature variation with electric field intensity at flow rate of 1.6 ml/h.

The electric field intensity has an important influence on the highest temperature of flame. Figure 10 shows the highest temperature variation with electric field intensity at the flow rate of 1.6 ml/h. With the increase of electric field intensity, stronger electric field can produce stronger ion wind, which can move evaporation particles escaped from the nozzle of the tube into the combustion zone to improve the combustion efficiency. This results in higher frame at the highest temperature. As the electric field intensity arrives at its threshold, the highest flame temperature decreases with increasing electric field intensity. The main reason is that ion wind of electric filed is so strong that more combustion particles can be moved out of the combustion zone to degrade the combustion efficiency when the fuel achieves full burning. Maximum value of highest flame temperature and its corresponding electric field intensity are presented in Table 3. As the inner diameter of tube is smaller, the highest temperature can reach maximum value faster, and its corresponding electric field intensity is also lower. It implies that microscale effect and ion wind of electric field can improve fuel combustion efficiency.

## Conclusions

In this paper, the laminar flow diffusion combustion characteristics of microscale liquid biobutanol in electric field were studied. Both in-lab experiment and numerical simulation suggest microscale effect and electric field have significant impacts on microscale combustion. According to the results of this study, we found:

1. Numerical simulation is able to measure the micro scale flame structure size and temperature. Numerical simulation results of height, width and temperature show a high consistency with the experimental results.
2. The results of experimental study show that microscale effect and electric field have a strong effect on the quenching flow rate of microscale flame. With the increase of DC voltage, the quenching flow rate first decreases and gradually increases after reaching its low peak value.
3. The results of numerical simulation show that traditional gas jet diffusion flame theory is not suitable to explain the micro scale flame for smaller tube, because of microscale effect and electric field. In addition, with the increase of electric field intensity, the flame height increases at first and then decreases. Moreover, the maximum flame height for smaller tube can be obtained faster.
4. As the liquid biobutanol flow rate increases, the highest temperature of flame increases almost linearly. At the same time, the electric field can destroy the complete combustion balance and degrade the flame temperature for smaller tube, but it can improve the combustion efficiency in larger tube due to the incomplete combustion. The highest flame temperature increases first and then decreases with the increase of electric field intensity. As the inner diameter of tube is smaller, the highest temperature can reach its maximum value faster.

## Additional Information

**How to cite this article**: Xu, T. *et al*. Effects of electric field on micro-scale flame properties of biobutanol fuel. *Sci. Rep.* **6**, 32938; doi: 10.1038/srep32938 (2016).

## Figures and Tables

**Table 1:** Parameters of biobutanol.

Purity	Density	Boiling Point	Spontaneous temperature	Low calorific value	Inflammability limit(volume)	viscosity
≥99.0%	0.81 g/cm^3^	117.25 °C	385 °C	33.1 MJ/kg	1.4%~11.2%	2.63 mm^2^/s

^*^Note: the fuel is supplied by Chengdu Lianhe Chemical Medicine Co., Ltd.

**Table 2:** Comparison of Microflame Characteristic Parameters.

			0 V		4000 V	
DC voltage			H/mm	T_A_/K	H/mm	T_A_/K
Experiment	tube1	0.9 ml/h	4.57	1528	4.60	1518
		1.4 ml/h	6.41	1496	8.17	1483
	tube2	1.8 ml/h	3.72	1428	4.09	1430
		2.4 ml/h	4.65	1413	5.13	1419
Numerical simulation	tube1	0.9 ml/h	4.80	1551	5.02	1535
		1.4 ml/h	6.78	1525	8.78	1507
	tube2	1.8 ml/h	3.84	1454	4.35	1462
		2.4 ml/h	4.99	1432	5.44	1445
Deviation	tube1	0.9 ml/h	5.03%	1.51%	9.13%	1.12%
		1.4 ml/h	5.77%	1.94%	7.47%	1.62%
	tube2	1.8 ml/h	3.23%	1.68%	6.36%	2.24%
		2.4 ml/h	7.31%	1.35%	6.04%	1.83%

**Table 3:** Maximum value of highest flame temperature and its corresponding electric field intensity.

Parameter	electric field intensity	maximum value of highest temperature
tube 1	60 V/mm	1679.6 K
tube 2	130 V/mm	1474.4 K

## References

[b1] Epstein A. H. & Senturia S. D. Macro Power from Micro Machinery. Science 276, 1211 (1997).

[b2] Spadaccini C. M. . High power density silicon combustion systems for micro gas turbine engines. J Eng Gas Turb Power 125, 709–719 (2003).

[b3] Mehra A. . A six-wafer combustion system for a silicon micro gas turbine engine. J Microelectromech S 9, 517–527 (2000).

[b4] Yang W. M. . Microscale combustion research for application to micro thermophotovoltaic systems. Energ Convers Manage 44, 2625–2634 (2003).

[b5] Dunn-Rankin D., Leal E. M. & Walther D. C. Personal power systems. Prog Energ Combust 31, 422–465 (2005).

[b6] Hussain T., Markides C. N. & Balachandran R. Flame Dynamics in a Micro-Channeled Combustor. Aip Conf Proc 1642, 130–137 (2015).

[b7] Loire S., Mezic I. & Fonoberov V. A. Combustion of Methane in Microchannels. Imece 2009: Proceedings of the Asme International Mechanical Engineering Congress and Exposition, Vol 12, Pts a and B, 719–723 (2010).

[b8] Maruta K. Flame Chromatography: Toward Fuel Indexing Based on Multiple Weak Flames in a Meso-Scale Channel with a Prescribed Temperature Profile. *Proceedings of the Asme 9th International Conference on Nanochannels, Microchannels and Minichannels 2011, Vol 2*, 593–598 (2012).

[b9] Yoshimoto T., Ikeda M., Kinoshita K., Kato Y. & Takagi T. Stability limits and behaviors of the diffusion flame. Combustion Science and Technology in Asia-Pacific Area: Today and Tomorrow, 109–112 (2003).

[b10] Fujiwara K. & Nakamura Y. Experimental study on the unique stability mechanism via miniaturization of jet diffusion flames (microflame) by utilizing preheated air system. Combust Flame 160, 1373–1380 (2013).

[b11] Irani A., Saediamiri M., Saidi M. S., Saidi M. H. & Shafii M. B. One-Dimensional Numerical Investigation of a Cylindrical Micro Combustor. *Ht2009: Proceedings of the Asme Summer Heat Transfer, Vol 3*, 115–123 (2009).

[b12] Jiang D. Y., Yang W. M. & Teng J. H. Entropy generation analysis of fuel lean premixed CO/H-2/air flames. Int J Hydrogen Energ 40, 5210–5220 (2015).

[b13] Z. Ren, H. Yang & T. Lu Effects of small-scale turbulence on NOx formation in premixed flame fronts. Fuel 115, 241–247 (2014).

[b14] Chein R. Y., Chen Y. C. & Chung J. N. Numerical study of methanol-steam reforming and methanol-air catalytic combustion in annulus reactors for hydrogen production. Applied Energy 102, 1022–1034 (2013).

[b15] Sadasivuni V. & Agrawal A. K. A novel meso-scale combustion system for operation with liquid fuels. P Combust Inst 32, 3155–3162 (2009).

[b16] Ferrante L., Miccio M., Solimene R. & Miccio F. An investigation on low-temperature fluidized combustion of liquid fuels. *Proceedings of the 18th International Conference on Fluidized Bed Combustion*, 433–441 (2005).

[b17] Ban H., Venkatesh S. & Saito K. Convection-Diffusion Controlled Laminar Micro Flames. Journal of Heat Transfer 116, 954–959 (1994).

[b18] Nakamura Y., Kubota A., Yamashita H. & Saito K. B13-118 Near extinction flame structure of micro-diffusion flames. *International Symposium on Micro-Mechanical Engineering : ISMME* **2003**, 163–170 (2003).

[b19] Matta L. M., Neumeier Y., Lemon B. & Zinn B. T. Characteristics of microscale diffusion flames. P Combust Inst 29, 933–939 (2002).

[b20] Spadaccini C. M., Zhang X., Cadou C. P., Miki N. & Waitz I. A. Preliminary development of a hydrocarbon-fueled catalytic micro-combustor. Sensors and Actuators A: Physical 103, 219–224 (2003).

[b21] Zhong Bei-Jing H. Z.-K. Numerical simulation of catalytic combustionof CH4 in micro-scale. Journal of Engineering Thermophysics 24, 173–176 (2003).

[b22] Norton D. G. & Vlachos D. G. Combustion characteristics and flame stability at the microscale: a CFD study of premixed methane/air mixtures. Chemical Engineering Science 58, 4871–4882 (2003).

[b23] Cheng T. S. . Experimental and numerical investigation of microscale hydrogen diffusion flames. P Combust Inst 30, 2489–2497 (2005).

[b24] Kyritsis D. C., Roychoudhury S., McEnally C. S., Pfefferle L. D. & Gomez A. Mesoscale combustion: a first step towards liquid fueled batteries. Experimental Thermal & Fluid Science 28, 763–770 (2004).

[b25] Pham T. K., Dunn-Rankin D. & Sirignano W. A. Flame structure in small-scale liquid film combustors. P Combust Inst 31, 3269–3275 (2007).

[b26] Balthasar M., Mauss F. & Wang H. A computational study of the thermal ionization of soot particles and its effect on their growth in laminar premixed flames. Combust Flame 129, 204–216 (2002).

[b27] Saito M., Sato M. & Sawada K. Variation of flame shape and soot emission by applying electric field. Journal of Electrostatics 39, 305–311 (1997).

[b28] Arai M., Saito M. & Arai T. Control of soot emitted from acetylene diffusion flames by applying an electric field. Combustion & Flame 119, 356–366 (1999).

[b29] Gan Y., Xue F. & Yang Z. Experimental study on the diffusion flame from small ceramic tube. *2010 Asia-Pacific Power and Energy Engineering Conference*, 1–4 (2010).

[b30] Yang Z., Xu T. & Gan Y. Experimental study on the diffusion flame using liquid ethanol as fuel in mini-scale. *Proceedings of the ASME Micro/Nanoscale Heat Transfer International Conference*, 853–857 (2008).

[b31] Liang Y. Z., Feng X. & Hua G. Y. Experimental study of small jet diffusion flame of alcohol with ceramic tube as burner. Journal of Thermal Science & Technology 7, 367–372 (2008).

[b32] Xu T. . Experimental and Numerical Simulation Study of the Microscale Laminar Flow Diffusion Combustion of Liquid Ethanol. Industrial & Engineering Chemistry Research 52, 8021–8027 (2013).

[b33] Calcote H. F. Electrical properties of flames1Burner flames in transverse electric fields. Symposium on Combustion & Flame & Explosion Phenomena 3, 245–253 (1949).

[b34] Calcote H. F. & Pease R. N. Electrical Properties of Flames. BurnerFlames in Longitudinal Electric Fields. Ind.eng.chem 43, 2726–2731 (1951).

[b35] Lapp M. & Rich J. A. Electrical Conductivities of Seeded Flame Plasmas in Strong Electric Fields. Physics of Fluids (1958–1988) 6, 806–816 (1963).

[b36] Popov V. A. & Sheklein A. V. Spectroscopic investigation of a plane methane-air flame in an electric field. Combustion Explosion & Shock Waves 1, 58–59 (1965).

[b37] Sánchez-Sanz M., Murphy D. C. & Fernandez-Pello C. Effect of an external electric field on the propagation velocity of premixed flames. P Combust Inst 35, 3463–3470 (2015).

[b38] Turns S. R. An Introduction to Combustion. Concepts and Applications. (Mcgraw-Hill Publ.Comp., 2000).

